# YEARS clinical decision rule for diagnosing pulmonary embolism: a prospective diagnostic cohort follow-up study in primary care

**DOI:** 10.1136/bmjopen-2024-091543

**Published:** 2025-02-06

**Authors:** Rosanne van Maanen, Hannah M la Roi-Teeuw, Frans H Rutten, Melchior Nierman, Frederikus A Klok, Menno V Huisman, Tuur Egbers, Jeanet Blom, Karel Moons, Geert-Jan Geersing

**Affiliations:** 1 Julius Center for Health Sciences and Primary Care, University Medical Center Utrecht, Utrecht, The Netherlands; 2 Department of Thrombosis, Atalmedial Medical Diagnostics Centers, Amsterdam, The Netherlands; 3 Department of Thrombosis and Hemostasis, Leiden University Medical Center, Leiden, The Netherlands; 4 Center for Thrombosis and Hemostasis (CTH), Johannes Gutenberg University Mainz, Mainz, Germany; 5 Department of General Practice and Nursing Science, Utrecht University, Utrecht, The Netherlands; 6 Leiden University Medical Center, Leiden, The Netherlands; 7 Department of Epidemiology, Julius Center for Health Sciences and Primary Care, Utrecht, The Netherlands

**Keywords:** Primary Healthcare, Thromboembolism, Cardiology

## Abstract

**Abstract:**

**Objectives:**

The Wells rule is often used in primary care to rule out pulmonary embolism (PE), but its efficiency is low as many referred patients do not have PE. In this study, we evaluated in primary care an alternative and potentially more efficient diagnostic strategy—the YEARS algorithm; a simplified three-item version of the Wells rule combined with a pretest probability adjusted D-dimer interpretation.

**Design:**

In this comprehensive prospective diagnostic validation study, primary care patients suspected of PE were enrolled by their general practitioner. All three YEARS items were collected in addition to D-dimer results, and patients were followed for 3 months to establish the final diagnosis.

**Setting:**

Primary care in the Netherlands.

**Participants:**

753 patients with suspected acute PE were included. Five patients (0.7%) were lost to follow-up.

**Main outcome measures:**

Failure rate (number of PE cases among patients classified by the algorithm as ‘PE ruled-out’) and efficiency (fraction of patients classified as ‘PE probable/further imaging needed’).

**Results:**

Prevalence of PE was 5.5% (41/748 patients). In total, 603 patients were classified as ‘PE ruled-out’ by the YEARS algorithm (532 with zero YEARS items and a D-dimer<1000 ng/mL and 71 with≥1 positive YEARS item and a D-dimer<500 ng/mL), resulting in an efficiency of 80.6% (603/748 patients, 95% CI 77.6% to 83.4%). Of these patients, three patients had a diagnosis of non-fatal PE during 3 months follow-up, all three with zero YEARS items and D-dimer between 500 and 1000 ng/mL, resulting in an overall diagnostic failure rate of 0.50% (3/603 patients, 95% CI 0.13% to 1.57%). In the patients categorised as ‘imaging needed’ (n=145), a total of 38 (26.2%) were indeed diagnosed with PE.

**Conclusions:**

Our study suggests that acute PE can be safely ruled out in 80% of patients by the YEARS algorithm in a primary care setting, while only 20% of patients required referral to hospital care for imaging tests. In those classified as ‘imaging needed’, PE was present in about one in every four patients, demonstrating a high detection proportion.

**STRENGTHS AND LIMITATIONS OF THIS STUDY**

- This study included a large sample of patients suspected of pulmonary embolism (PE) in primary care to validate the YEARS criteria for the first time in this clinical domain.
- Two different routes of patient recruitment were used that may differ in terms of patient selection and subsequent patient referral patterns, potentially influencing our results.
- The study was not powered or designed to compare the YEARS criteria with other diagnostic strategies for suspected PE, such as for instance the Wells rule.

## Introduction

 The diagnostic management of patients suspected of pulmonary embolism (PE) in primary care is challenging. The non-specific symptoms of PE may mimic other, less severe diagnoses such as myalgia or a respiratory tract infection, but also other life-threatening events such as acute coronary syndrome.^1 2^ Considering the potential morbidity and mortality of PE, prompt and adequate decisions about further testing (eg, D-dimer and radiological imaging) and referral to hospital care of those suspected of PE are pivotal.^3 4^ On the other hand, over-testing of patients with a low risk of PE can also lead to harm, for instance, due to exposure to radiation, or increasing the risk of finding small subsegmental PE’s that are not clinically relevant).^5^ Moreover, refraining from imaging tests if not indicated has a positive climate impact as well as it reduces the use of natural and energy resources, thereby CO^2^ emissions, positively contributing to human health as well.^6^ Therefore, general practitioners (GPs) use clinical decision rules to stratify patients suspected of PE as either at high risk—warranting swift referral for subsequent diagnostic testing—or at low risk, in which case watchful waiting is adequate. In the past decades, several clinical decision rules to classify PE-suspected patients have been developed and validated.^7^,^9^

The Wells rule is currently implemented as standard-of-care for suspected patients with PE in primary care.^10^ In a previous validation study including primary care patients, the application of the Wells score resulted in a referral rate of 54% (ie, an efficiency of 46% for which referral was not needed) with an adequately safe failure (false negative) rate of 1.5%.^11^ Still, most referred patients are ultimately not diagnosed with PE. Notably elderly patients and patients with comorbidities may often have D-dimer values between 500 and 1000 ng/mL without having a PE according to CT pulmonary angiography (CTPA).^12^ Besides, we previously demonstrated that the Wells rule is frequently incorrectly applied in real-world day-to-day primary care, which is associated with an even lower efficiency and, importantly, also a higher failure rate.^13^ Easier algorithms thus are highly awaited. Such tools should preferably (1) better incorporate flexible D-dimer thresholds to adjust for comorbidity and/or pretest probability and (2) are less sensitive to incorrect application.

The YEARS algorithm is such a simplified clinical decision rule that was developed and validated in hospital settings.^14^,^16^ The algorithm is easier and more efficient than the Wells score, using only three items the physician needs to score—clinical signs of deep venous thrombosis (DVT), haemoptysis and PE considered the most likely diagnosis—to determine pretest PE risk stratum, indicating D-dimer testing in both strata, and adopting a higher threshold (1000 ng/mL) for the low pretest PE risk stratum. In hospital settings, the application of the YEARS algorithm resulted in a 14% reduction of CTPAs compared with a fixed D-dimer threshold, while PE diagnosis was missed in only 0.6% of patients (well below the internationally accepted and widely used safety threshold of 3%).^11 15 17^ An individual patient data meta-analyses using retrospective data already suggested that the YEARS algorithm might also be safe and more efficient than the Wells rule in primary care.^18^ However, before widespread implementation and incorporation into primary care guidelines, prospective evaluation is pivotal to confirm or refute the safety of the YEARS algorithm in primary care. Such evidence is currently lacking. This large-scale prospective diagnostic cohort study therefore aimed to evaluate the diagnostic safety and efficiency of the YEARS algorithm in primary care.

## Methods

Detailed methods of this prospective diagnostic validation study are described in the previously published study protocol.^19^ The Standards for Reporting Diagnostic Accuracy guidelines were followed where appropriate.

### Participants

Eligible were all patients aged 18 years or older presenting in participating GP practices spread across the Netherlands with signs and symptoms that were suspicious of acute PE, defined as (sub)acute onset of unexplained shortness of breath with or without chest discomfort such as pleuritic chest pain. We deliberately did not dictate more specifically when and in what type of patients PE should be suspected in primary care, to mimic daily primary care as much as possible. We did however provide the following exclusion criteria: (1) current treatment with therapeutic doses of vitamin K antagonists, low-molecular-weight heparin or a direct oral anticoagulant; (2) life expectancy less than 1 month estimated by the GP; and (3) pregnancy until 6 weeks after delivery. All included patients provided written informed consent.

### Recruitment and diagnostic work-up

Originally, this study was set up as a multicentre study in 50 participating GP practices with patients being managed according to the YEARS algorithm (we refer to this route as the ‘prospective management route’, see study protocol). Consecutive patients visiting their GP with suspected PE were asked for informed consent. Next, the GP scored the three YEARS items, collected additional data and performed a D-dimer test. Different quantitative D-dimer assays were used via the laboratories of the participating regions. GPs were instructed to manage these patients according to the YEARS algorithm, that is, to only refer patients without YEARS items and a D-dimer above 1000 ng/mL, or with one or more YEARS items and a D-dimer above 500 ng/mL, for diagnostic CTPA imaging in the hospital. However, the final decision for referral and any subsequent care was left at the discretion of the GP (as in real-world clinical practice). Patients were included via this prospective management route from November 2018 to November 2022.

Because of lagging patient accrual (largely) due to COVID-19 and its associated impact on healthcare and healthcare research, it was decided to expand the study in October 2021 by a new easier-to-use inclusion route for GPs which was blended into the digital system that GPs use for D-dimer test ordering at the medical diagnostic centre in the wider Amsterdam region (we refer to this route as the ‘prospective observational route’). If the GP was ordering a D-dimer, the digital system prompted a question whether this D-dimer test was performed for a patient with suspected PE. Participating GPs consented to this method and had the possibility to ignore this automated digital message. Yet, if the GP then answered ‘yes’, the system automatically showed information about the YEARS algorithm (including its proposed flexible D-dimer thresholds), and our study aims and prompted the three YEARS-items, and the GP was asked to answer them before completing the D-dimer request. Hence, GPs were not influenced by D-dimer findings when scoring the YEARS items, including the item of ‘PE most likely diagnosis’ that potentially is most influenced by D-dimer findings (yet in our study, this was thus by design not possible). Subsequently, the score of the three YEARS items was gathered by the medical diagnostic centre, and eligible patients received a letter to ask for consent for study participation. Only on consent, the patients’ sex, age, YEARS items, and D-dimer result (all tested with the Innovance D-dimer assay, point-of-care assays were not available during patient inclusion, thus also not used in this study) were made available to the researchers. The care providing GPs in this route were not formally instructed to manage patients according to the YEARS algorithm as was the case in the original developed route. Rather, referral decisions in this route were left at the discretions of the treating GP. Also, given that this study was partly conducted during the COVID-19 pandemic that already put a strain on primary healthcare, we did not ask participating GPs to collect additional patient characteristics other than what was strictly needed to answer our research question. Patients were included via this prospective observational route from September 2021 to March 2023. The Medical Ethical Committee Utrecht discussed the amendment of this additional recruitment route and judged it (according to Dutch law) to be exempt from formal additional review as all study procedures in this route were part of routine medical care.

### Endpoints

In both inclusion routes, all included patients were followed up prospectively for 3 months to assess whether PE was indeed diagnosed, in line with other studies performed in the field of diagnosing PE.^12 15^ As GPs in the Netherlands have a gatekeepers’ role with listed patients for whom they deliver integrated and continuity of care, this approach of endpoint collection was shown to be valid in previous studies from our group.^11^,^13^ In short, 3 months after the inclusion date, questionnaires were sent to the GPs of all enrolled participants (from both inclusion routes) to verify the diagnostic outcomes. In this follow-up questionnaire, the GPs were asked whether PE was diagnosed and, if not, whether alternative diagnoses were present or identified during follow-up (options: myalgia, pneumonia, chronic obstructive pulmonary disease/asthma, cardiological, heart failure, other or unknown). For referred patients, this information was retrieved from hospital letters sent to the GP (which is a standard procedure in the Dutch healthcare system). For not referred patients, the follow-up period in primary care was used to assess a final diagnosis by the GP. A diagnosis of PE at baseline or during the 3 months of follow-up was defined as at least one of the following: (1) a CTPA demonstrating a filling defect in a central, segmental or lobular pulmonary artery or a subsegmental filling defect that required anticoagulant therapy, or (2) a ventilation/perfusion lung scan suggestive of PE, or (3) a pulmonary angiogram demonstrating an intraluminal filling defect, or (4) fatal PE following ISTH definitions or (5) DVT was confirmed with ultrasonography of the leg in patients (initially) suspected of PE. In patients with uneventful follow-up, so without fulfilling any of the five above-described confirmations of PE diagnosis, PE was considered absent. All diagnostic work-up after referral was performed as routine care, with care providers fully aware of the patients’ clinical symptoms, medical history and D-dimer results (endpoint assessors were not blinded).

### Data analysis

Given the low proportion of patients lost to follow-up, only patients with available follow-up were analysed (complete case analysis). Patient characteristics, the presence or absence of the three YEARS items and the remaining Wells items (only for the prospective management route), median (IQR) D-dimer values and PE prevalence were reported for the total study cohort and stratified by inclusion route. Based on 2×2-tables, sensitivity, specificity, failure rate and efficiency were calculated with corresponding 95% CIs using the exact method. Efficiency was defined as the proportion of patients classified as ‘PE ruled out’ according to the YEARS algorithm (ie, the proportion of patients that should not be referred for diagnostic imaging) among the total study population. The failure rate was defined as the proportion of patients with a PE diagnosis within 3 months of follow-up among these patients classified as ‘PE ruled out’. Failure rate and efficiency were also calculated stratified by inclusion route. Lastly, we described in detail all patients with a false-negative ‘PE ruled-out’ classification (failure cases) and deceased patients. Quantitative data analyses were performed in R V.4.0.3.

### Sample size calculation

Details on sample size calculation are described in the study protocol.^19^ Briefly, sample size was based on the internationally accepted safety upper margin of 3% for missed PE cases (false negatives),^11 20 21^ using an estimated false-negative rate and proportion of patients in the ‘PE-ruled out’ classification by the YEARS algorithm from previous studies,^11 15^ and assuming 10% loss to follow-up. This led to a targeted sample size of 750 patients of which at least 300 patients should be categorised as ‘PE ruled out’.

### Patient and public involvement

Patients were not directly involved in the development of the study design and protocol.

## Results

**Figure 1:**
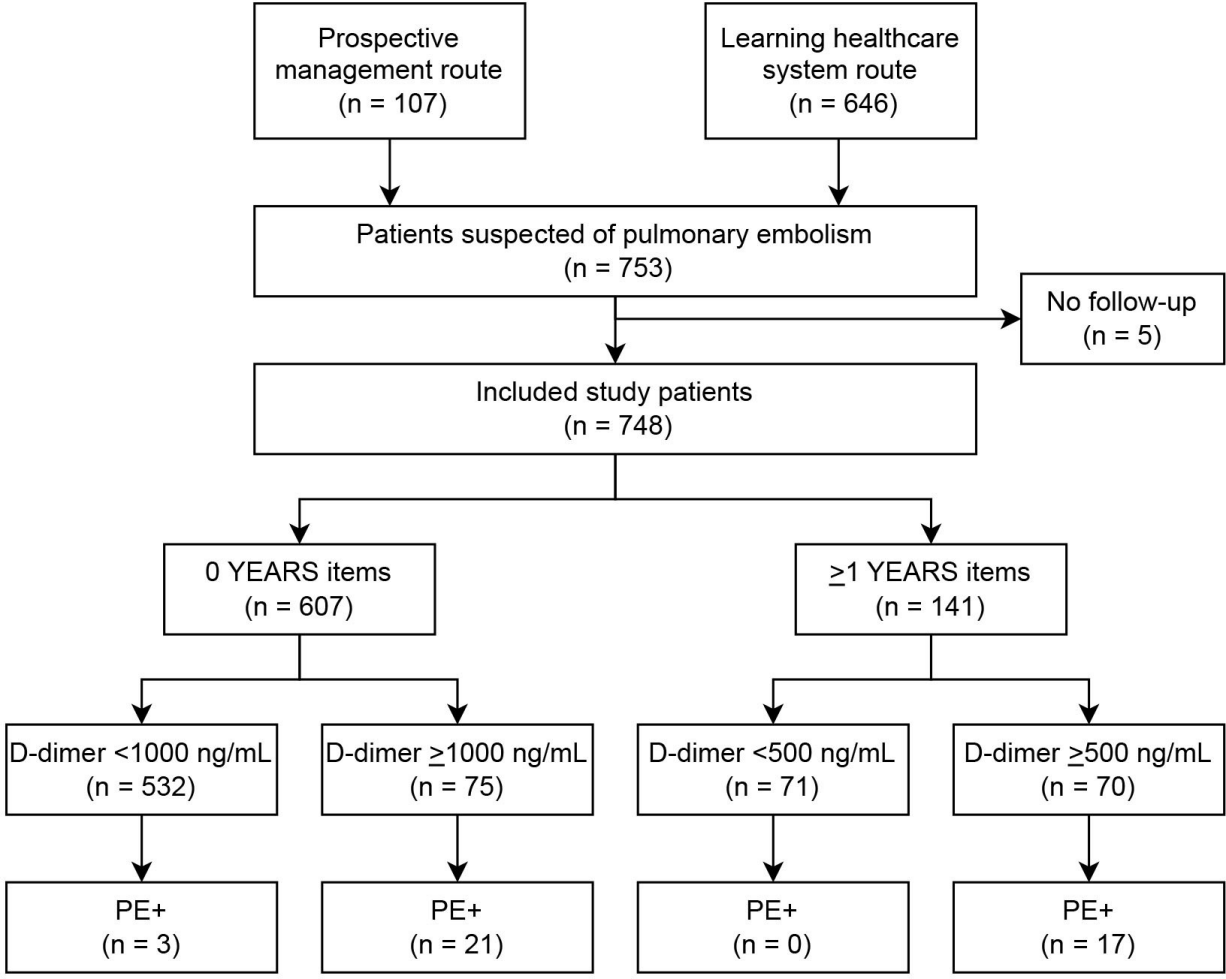
Flowchart of included patients. PE, pulmonary embolism.

In total, 753 patients were included of which 107 consecutive patients with clinically-suspected PE were included via the prospective management route. In the prospective observational route, 1517 patients with suspected PE were identified of whom 646 patients (43%) consented for study participation (figure 1). In only 5 (0.7%) patients, the GP did not return the follow-up form with the final diagnosis, and these patients were excluded from our analysis. The total study population thus consisted of 748 patients.

### Patient characteristics

The clinical characteristics of the 748 analysed patients are shown in table 1. The median age was 55 (IQR 41.75–66) years and 70% were women. The YEARS item, ‘PE most likely diagnosis’ was more often scored to be present in the prospective management route (26.2%), compared with the prospective observational route (10.0%). The other two YEARS items (ie, clinical signs of DVT and haemoptysis) were scored to be present in 4.7% and 5.6% in the prospective management route and 2.3% and 5.1% in prospective observational route, respectively. The prevalence of PE was 5.5%. In patients not diagnosed with PE, myalgia was the most common alternative diagnosis (diagnosed in 25% of patients without PE). Among these patients, those categorised as ‘Imaging needed’ according to the YEARS algorithm were more likely to have other alternative diagnoses such as pneumonia or cardiological diagnoses compared with those categorised as ‘PE ruled out’ (table 2).

**Table 1:** Clinical characteristics of 748 included patients stratified per inclusion route

	Missing proportion	Prospective management route (n=107)	Prospective observational route (n=641)	Total study population (n=748)*
Median age, years (IQR)	0	52 (34–62.5)	55 (42–66)	55 (41.75–66)
Women, n (%)	3 (0.4)	78 (72.9)	443 (69.1)	521 (69.7)
Median D-dimer, ng/mL (IQR)	0	410 (220–610)	380 (214–673)	382 (216–669)
PE most likely diagnosis, n (%)	0	28 (26.2)	64 (10.0)	92 (12.3)
Clinical signs of DVT, n (%)	0	5 (4.7)	15 (2.3)	20 (2.7)
Haemoptysis, n (%)	0	6 (5.6)	33 (5.1)	39 (5.2)
Heart rate>100/min, n (%)	641 (85.7)	31 (29.0)	n.a.	n.a.
Immobilisation/surgery<1 month, n (%)	643 (86.0)	6 (5.6)	n.a.	n.a.
Active malignancy, n (%)	641 (85.7)	0	n.a.	n.a.
History of VTE, n (%)	641 (85.7)	6 (5.6)	n.a.	n.a.
Final PE diagnosis, n (%)	0	8 (7.5)	33 (5.1)	41 (5.5)

* Percentages were calculated in patients without missing data on that variable.

DVT, deep venous thrombosis; n.anot assessedPEpulmonary embolism VTE, venous thromboembolism

**Table 2:** Alternative diagnoses of 707 included patients without pulmonary embolism stratified by YEARS classification

	YEARS‘PE ruled out’ (n=600)	YEARS‘imaging needed’ (n=107)	Total (n=707)
Myalgia, n (%)	162 (27.0)	15 (14.0)	177 (25.0)
Pneumonia, n (%)	48 (8.0)	18 (16.8)	66 (9.3)
COPD/asthma, n (%)	40 (6.7)	9 (8.4)	49 (6.9)
Cardiological, n (%)	21 (3.5)	9 (8.4)	30 (4.2)
Heart failure, n (%)	4 (0.7)	6 (5.6)	10 (1.4)
Other, n (%)	214 (35.7)	46 (43.0)	260 (36.8)
Unknown, n (%)	146 (24.3)	18 (16.8)	164 (23.2)

COPDchronic obstructive pulmonary diseasePEpulmonary embolism

### Safety, efficiency and diagnostic accuracy

Table 3 shows the results of the study. A total of 603 patients were categorised as ‘PE ruled out’ according to the YEARS algorithm, with either no YEARS items and a D-dimer below 1000 ng/mL (n=532) or one or more YEARS items and a D-dimer below 500 ng/mL (n=71). In these 603 patients, PE would be considered ruled-out and referral would not be recommended according to the YEARS algorithm. This group thus compromises of 532 patients with a YEARS score of 0 points and a D-dimer below 1000 ng/mL—398 patients with a D-dimer below 500 ng/mL (no missed PE cases) and 134 patients with a D-dimer between 500 and 1000 ng/mL (failure rate 2.24% (95% CI 0.58% to 6.90%)—and 71 patients with a YEARS score of 1 and a D-dimer below 500 ng/mL (no missed PE cases). This resulted in an overall efficiency of 80.6% (95% CI 77.6% to 83.4%) (table 4). In the patients categorised as ‘imaging needed’ (n=145), a total of 38 (26.2%) were diagnosed with PE. Of the 603 patients categorised as ‘PE ruled out’ at baseline, three patients had a non-fatal PE diagnosed within 3 months of follow-up, resulting in a failure rate of 0.50% (95% CI 0.13% to 1.57%) (table 4). There were no clear differences in efficiency or failure rate between both inclusion routes (table 4).

**Table 3:** Contingency table

	PE diagnosis	No PE	Total
YEARS ‘Imaging needed’	38	107	145
YEARS ‘PE ruled out’	3	600	603
Total	41	707	748

PEpulmonary embolism

**Table 4:** Stratified analysis of primary outcomes in different inclusion routes

Inclusion route	Prevalence PE, %	Failure rate (95% CI), %	Efficiency (95% CI), %
Management route (n=107)	7.5	0.00 (0.00 to 5.51)	77.6 (68.3 to 84.8)
Learning healthcare system route (n=641)	5.1	0.58 (0.15 to 1.82)	81.1 (77.8 to 84.0)
Total study population (n=748)	5.5	0.50 (0.13 to 1.57)	80.6 (77.6 to 83.4)

PEpulmonary embolism

The overall sensitivity of the YEARS algorithm in this study was 0.93 (95% CI 0.79 to 0.98) and the specificity 0.85 (95% CI 0.82 to 0.87). The positive predicted value was 26.2% (95% CI 22.6% to 30.1%), and the negative predictive value was 99.5% (95% CI 98.5% to 99.8%).

### Failure cases

In three patients, a PE diagnosis was missed at baseline. These three patients were included via the prospective observational route. All three had D-dimer values between 500 and 1000 ng/mL had a YEARS score of 0 points and were diagnosed by diagnostic imaging, either directly or during follow-up. Patient 1 was a male patient of 55 years with no relevant medical history. His YEARS score was 0, with a D-dimer level of 712 ng/mL at baseline. Initially, he was not referred to the emergency department; 1 month after baseline, he was submitted to the hospital and was diagnosed with subsegmental PE. Patient 2 was a male patient of 59 years with an ablation for atrial flutter 1 year prior to his visit to the GP for suspected PE. His YEARS score was 0, with a D-dimer level of 795 ng/mL. At baseline, his GP referred him to the emergency department because of worsening pleuritic chest pain. CTPA showed segmental, non-fatal PE. Finally, patient 3 was a female patient of 72 years old. She was diagnosed with myoclonus dystonia and received a deep brain stimulator 1 year prior to her consultation for suspected PE with her GP. Her YEARS score was 0, with a D-dimer of 834 ng/mL. Her GP referred her to the cardiologist for radiating chest pain, directly at the initial suspicion of PE. CTPA revealed segmental, non-fatal PE, diagnosed by the cardiologist on initial referral.

### Mortality

Three out of the 748 patients died during follow-up. All three were included via the prospective observational route, had no YEARS items and a D-dimer value below 1000 ng/mL. None of them was diagnosed with PE. Their recorded causes of death were lung cancer (n=2) and euthanasia (n=1). According to the proposed classification by the International Society of Thrombosis and Haemostasis, these causes of death would all be classified as C (cause of death other than PE), so not being PE related.^22^

## Discussion

In this comprehensive prospective diagnostic validation study of the YEARS algorithm in primary care, about 80% of patients suspected of PE were classified as ‘PE ruled out’ according to the YEARS algorithm so that a hospital referral for CTPA was not indicated. The diagnostic PE failure rate in this group was 0.50% (95% CI 0.13% to 1.57%), and none of the failure cases died of PE. Moreover, in the remaining 20% of patient classified as ‘imaging needed’, a referral indeed was warranted given that PE was present in about a quarter of these patients. Following guidance from the International Society of Thrombosis and Haemostasis on diagnostic studies in the field of PE, this indicates that the YEARS algorithm is a safe algorithm to rule-out PE in the primary care setting, importantly at a very high efficiency and with a high diagnostic detection proportion in the referred patients.^23^

### Comparison with existing literature

The failure rate of the YEARS algorithm in primary care in our current study is comparable to those from another prospective validation study of the YEARS algorithm in secondary care.^15^ However, the 80% efficiency is much higher than the 55% efficiency from a recent, although retrospective, individual patient data meta-analysis including more than 3000 primary care patients suspected of PE.^18^ It is also higher than the reported 46% efficiency for the Wells rule for PE.^11^ This might be caused by the rather low prevalence of PE (5.5%) in our study compared with older primary care studies (prevalence of PE ranging from 12% to 28%).^11 12^ Although selection bias through exclusion of high-risk patients (according to the Wells rule or GPs’ gestalt) may have played a role in the prospective observational route in our study, PE prevalence was also low in the prospective management route (7.5%). Interestingly, this lowering of PE prevalence in suspected patients seems to be in line with a worldwide tendency to a lower threshold of suspicion of PE in the last decade.^24^ This may also be reflected in the proportion of patients in whom the subjective item ‘PE most likely diagnosis’ was positively scored. In our current dataset, this concerned 26% of patients in the prospective management route, which is substantially lower than in the YEARS validation study in the hospital setting (50%) or a study performed between 2014 and 2017 in primary care (56%).^11 15^ Although scoring this subjective item could be considered challenging, our study group has previously showed that when physicians assess PE as most likely diagnosis, the risk of PE increased threefold, interestingly irrespective of the clinical setting where patients are encountered.^25^ Furthermore, also as an exemplification of scoring this item ‘PE most likely’, recent evidence showed that most doctors are indeed currently more concerned about missing a PE diagnosis, which may lead to defensive diagnostic strategies.^26 27^ This increased tendency for referral is of concern, as it may unintentionally lead to over-testing for PE and subsequent harm to patients (eg, by radiation exposure or overtreatment of subsegmental PE of unknown clinical significance), a burden to the healthcare system and a climate impact that is potentially avoidable. In that respect, the currently observed high efficiency of the YEARS algorithm in our study suggests that implementing this strategy in primary care may help in preventing the burdens of over-testing and overdiagnosis of PE, while at the same time also safely rules-out PE in suspected patients.

### Strengths and limitations

To our knowledge, this is the first study that has prospectively collected data to evaluate the YEARS algorithm in patients with suspected PE in the primary care setting. Our results are representative of daily clinical practice given the pragmatic design and were consistent across two different inclusion routes. However, the current study should also be interpreted in the light of some limitations.

First, we have not directly compared the YEARS strategy to the Wells strategy by design. We refrained from an observational comparison within our data given the small sample of patients in the prospective management route for which data on Wells items were available. For patients included via the prospective observational route, unfortunately, we did not collect all (other) Wells rule items, to simplify and facilitate the participation of GPs via this route during the COVID-19 pandemic. However, to provide some comparison, in our previously published individual patient data meta-analysis, we retrospectively compared the Wells rule with the YEARS algorithm, also for primary care patients suspected of PE.^18^ In that analysis we found that both approaches can safely exclude PE in primary care, yet applying the YEARS algorithm was associated with a much higher efficiency. This high efficiency of using the YEARS algorithm, without hampering the safety of ruling out PE in primary care, is also one of our key findings in the current study.

Second, in contrast to our intended study design as a diagnostic management validation, we had to enrol most patients in this study from an additional inclusion route in which GPs did prospectively collect data yet were not asked to manage their patients according to the YEARS algorithm. Hence, data collection in these patients was prospective while the evaluation of YEARS in these patients is a ‘scenario analysis’, exemplifying on what the validity of the YEARS algorithm should have been if GPs managed all patients using the YEARS algorithm. The impact of this limitation is likely threefold. First, patients included via the prospective observational route may represent a lower risk group, potentially preselected using the Wells rule or clinical gestalt (as also reflected by the lower proportion of scored ‘PE most likely diagnosis’, 10% vs 26%), thus likely decreasing PE prevalence. To a relatively small extent, this indeed is what we observed in our data (5.1% for prospective observational route vs 7.5% for the management route). This might have potentially overestimated efficiency in our analysis. Second, on the same page, our findings are not generalisable to a higher PE risk/prevalence population, as we know from a previous individual patient data meta-analysis that with increasing PE prevalence the likelihood of missing PE also rises.^18^ Nevertheless, in that same publication, we showed that this only becomes problematic (ie, unsafe) if PE prevalence rises above 20%, indeed much higher than our overall observed PE prevalence of 5.5%. Moreover, likely, the clinical uncertainty is not in the group of patients at higher risk of having PE as GPs patients will refer these patients to an emergency department anyhow. Third, patients included via the prospective observational route with no YEARS items and a D-dimer between 500 and 1000 ng/mL may have been referred for diagnostic imaging despite low-risk (‘PE ruled out’) classification by the YEARS algorithm, which could have resulted in a higher rate of (sub)segmental PE with unknown clinical significance.^28^ Indeed, one of the three missed PE cases (included via the prospective observational route) was diagnosed with subsegmental PE.

Third, PEs might have been missed in all patients who were not referred for diagnostic imaging which may cause differential verification bias, yet such failure cases are likely of unknown clinical significance. Similarly, the decision to refer for imaging at least partly was dependent on the index test (in this case, the YEARS algorithm). Thus, the interpretation of our (combined) reference standard (essentially, the combination of imaging in those referred and clinical follow-up in those not referred) included information from our index test. This might lead to incorporation bias possibly overestimating diagnostic accuracy of our index test.^29^

Fourth, we cannot strictly confirm that none of the patients enrolled via the prospective observational route used anticoagulants; this would be a reason to refrain from using either the Wells or YEARS algorithm. Fifth, different types of D-dimer assays were used. Although these are all limitations from an experimental perspective, most of these issues are fully in line with real-world GP practice and may therefore render results more representative from a pragmatic perspective.

Altogether, these limitations should also stress the remaining uncertainty on whether the YEARS algorithm can safely be applied in daily primary care, based on only the findings from this current study, even though our findings are in line with the aforementioned (previously published) individual patient data meta-analysis retrospectively evaluating the safety and efficiency of the YEARS algorithm in more than 3000 primary care patients suspected of PE.^18^

### Implications for clinical practice and future research

Our data support the use of the YEARS algorithm to rule out acute PE without imaging in the primary care setting. The now established prospective observational route could facilitate further implementation of the YEARS algorithm. For example, after GPs have filled out the three YEARS items on ordering a D-dimer test, the laboratory could send the D-dimer result with the corresponding threshold and subsequent management recommendations. This could improve the correct use of the YEARS algorithm, which is important as we know from literature that incorrect use of the Wells rule is associated with an increased failure rate and reduced efficiency.^13^ Future pragmatic (cluster) randomised trials with a direct comparison between different strategies would provide better evidence on the difference in efficiency between clinical prediction rules.

### Conclusion

Our study, performed in a PE-suspected primary care population with a relatively low prevalence of PE, suggests that a ‘PE ruled out’ classification according to the YEARS algorithm can safely and efficiently exclude PE in the primary care setting and yield a high detection proportion in the referred patients.

## Data Availability

Data are available upon reasonable request.
