# Supplementary material for: YEARS clinical decision rule for diagnosing pulmonary embolism: a prospective diagnostic cohort follow-up study in primary care
Source: BMJ Open. 2025 Feb 6;15(2):e091543. doi: 10.1136/bmjopen-2024-091543 (PMC11800211; doi:10.1136/bmjopen-2024-091543)
Supplement: Supplementary file 1 [file bmjopen-2024-091543.reviewer_comments.pdf]

PEER REVIEW HISTORY

BMJ Open publishes all reviews undertaken for accepted manuscripts. Reviewers are asked to complete a checklist review form and are provided with free text boxes to elaborate on their assessment. These free text comments are reproduced below.

This paper was submitted to another journal from BMJ but declined for publication following peer review. The authors addressed the reviewers' comments and submitted the revised paper to BMJ Open. The paper was subsequently accepted for publication at BMJ Open.

ARTICLE DETAILS

Title (Provisional)

The YEARS clinical decision rule for diagnosing pulmonary embolism: a prospective diagnostic cohort follow-up study in primary care.

Authors

van Maanen, Rosanne; la Roi-Teeuw , Hannah M; Rutten, Frans H; Nierman, Melchior; Klok, Frederikus A; Huisman, Menno V; Egbers, Tuur; Blom, Jeanet; Moons, Karel; Geersing, Geert-Jan

---

VERSION 1 – REVIEW

**Reviewer**	**1**
**Name**	**Reviewer 1**
**Affiliation**	
**Date**	**22-Jul-2024**
**COI**	**No competing interest**

Comments not published pending reviewer's consent

**Reviewer**	**2**
**Name**	**Reviewer 2**
**Affiliation**	
**Date**	**22-Jul-2024**

**COI**	**No competing interest**

Comments not published pending reviewer's consent

**Reviewer**	**3**
**Name**	**Reviewer 3**
**Affiliation**	
**Date**	**22-Jul-2024**
**COI**	**No competing interest**

Comments not published pending reviewer's consent

VERSION 2 – REVIEW

**Reviewer**	**1**
**Name**	**Roy, Pierre-Marie**
**Affiliation**	**University Hospital of Angers, Emergency Medicine**
**Date**	**06-Nov-2024**
**COI review.**	**I understand and consent to the named publication of this**

The authors should be commended for this revised version of their article and for the thorough rebuttal they provided. They have thoughtfully addressed all remarks from both the editors and reviewers. Notably, they clarified the prospective assessment of the Years items in the LHS route and enriched the discussion section with key elements on the impacts of this significant protocol modification.

However, I have a few additional suggestions:

1. Terminology: For clarity, I recommend referring to the modified route as the “prospective observational route” or simply the “observational route” instead of the “LHS route.” This change would emphasize the primary difference between the “prospective management route” and the “prospective observational route.” Consider also whether the term “prospective” is essential in both labels.

2. Abstract: The two routes, along with their relative importance in terms of inclusions, should be mentioned in the abstract.

3. Table 1 - Clinical Characteristics: In describing the clinical characteristics of included patients (Table 1), results in the total study population (last column) should be provided only if they were assessed across both routes.

4. Table 2 - Alternative Diagnoses: Further explanation is needed regarding the alternative diagnoses listed in Table 2. Do these refer to other hypotheses considered by the GP (indicating a prospective assessment before knowing the D-dimer test result), or are they the final diagnoses retained by the GP for patients without pulmonary embolism?

5. Failure Cases: Could additional information be provided on failure cases, specifically regarding the signs and symptoms that raised suspicion of PE and details on the location and severity of PE in cases 2 and 3?

6. Conclusion Wording in the Abstract: I appreciate the measured tone in the main study conclusion, which reads, "Our study (…) suggests that …" This moderated language should also be used in the abstract's conclusion, rather than the stronger statement, "Acute PE was safely ruled out …, demonstrating a high detection proportion."

7. "What This Study Adds" Section: Similar moderation is recommended in the "What this study adds" paragraph. The phrase "in this large prospective diagnostic study in primary care, the YEARS algorithm proved safe and efficient" seems overly affirmative.

VERSION 2 - AUTHOR RESPONSE

Reviewer: 1
Dr. Pierre-Marie Roy, University Hospital of Angers, Université d'angers, UFR Santé

Comments to the Author:

The authors should be commended for this revised version of their article and for the thorough rebuttal they provided. They have thoughtfully addressed all remarks from both the editors and reviewers. Notably, they clarified the prospective assessment of the Years items in the LHS route and enriched the discussion section with key elements on the impacts of this significant protocol modification.

Answer: We thank the reviewer for his compliments.

However, I have a few additional suggestions:

1. Terminology: For clarity, I recommend referring to the modified route as the "prospective observational route" or simply the "observational route" instead of the "LHS route." This change would emphasize the primary difference between the

“prospective management route” and the “prospective observational route.” Consider also whether the term “prospective” is essential in both labels.

Answer: We now renamed the LHS route to ‘prospective observational route’, as suggested by this reviewer, throughout the paper.

2. Abstract: The two routes, along with their relative importance in terms of inclusions, should be mentioned in the abstract.

Answer: This has now been added to the strengths and limitations section of the abstract.

3. Table 1 - Clinical Characteristics: In describing the clinical characteristics of included patients (Table 1), results in the total study population (last column) should be provided only if they were assessed across both routes.

Answer: We agree, this has now been changed accordingly.

4. Table 2 - Alternative Diagnoses: Further explanation is needed regarding the alternative diagnoses listed in Table 2. Do these refer to other hypotheses considered by the GP (indicating a prospective assessment before knowing the D-dimer test result) or are they the final diagnoses retained by the GP for patients without pulmonary embolism?

Answer: It is the latter, final diagnoses as retained by the GP during follow-up for patients without PE. We tried to clarify this issue a bit more in the Methods section under the paragraph ‘Endpoints’, using track changes.

5. Failure Cases: Could additional information be provided on failure cases, specifically regarding the signs and symptoms that raised suspicion of PE and details on the location and severity of PE in cases 2 and 3?

Answer: This is added to the section on ‘Failure cases’. In case 2, the GP referred the patient directly to the ER because of worsening pleuritic chest pain. In case 3, the patient was referred to the cardiologist and PE was diagnosed by the cardiologist. In both cases, the segmental, non-fatal PE was diagnoses.

6. Conclusion Wording in the Abstract: I appreciate the measured tone in the main study conclusion, which reads, “Our study (…) suggests that …” This moderated language should also be used in the abstract’s conclusion, rather than the stronger statement, “Acute PE was safely ruled out …, demonstrating a high detection proportion.”

Answer: Good point, this wording has been changed accordingly, using track changes.

7. "What This Study Adds" Section: Similar moderation is recommended in the “What this study adds” paragraph. The phrase “in this large prospective diagnostic study in primary care, the YEARS algorithm proved safe and efficient” seems overly affirmative.

Answer: This section was removed upon recommendation by the BMJ Open editors as this section is not mandatory for BMJ Open.
